# Allosteric and ATP-Competitive MEK-Inhibition in a Novel Spitzoid Melanoma Model with a RAF- and Phosphorylation-Independent Mutation

**DOI:** 10.3390/cancers13040829

**Published:** 2021-02-16

**Authors:** Luca Hegedüs, Özlem Okumus, Elisabeth Livingstone, Marcell Baranyi, Ildikó Kovács, Balázs Döme, József Tóvári, Ágnes Bánkfalvi, Dirk Schadendorf, Clemens Aigner, Balázs Hegedüs

**Affiliations:** 1 Department of Thoracic Surgery, University Medicine Essen–Ruhrlandklinik, Tüschener Weg 40, 45239 Essen, Germany; luca.hegedues@rlk.uk-essen.de (L.H.); oezlem.okumus@stud.uni-due.de (Ö.O.); Clemens.Aigner@rlk.uk-essen.de (C.A.); 2 Department of Dermatology, University Medicine Essen, Esmarchstraße 14, 45147 Essen, Germany; Elisabeth.Livingstone@uk-essen.de (E.L.); dirk.schadendorf@uk-essen.de (D.S.); 3 2nd Department of Pathology, Semmelweis University, Üllői út 93, 1091 Budapest, Hungary; baranyi.marcell@med.semmelweis-univ.hu; 4 National Korányi Institute of Pulmonology, Pihenő út 1, 1122 Budapest, Hungary; kovaacs.ildi@gmail.com (I.K.); balazs.dome@meduniwien.ac.at (B.D.); 5 Department of Thoracic Surgery, National Institute of Oncology, Ráth György u. 7-9, 1122 Budapest, Hungary; 6 Department of Thoracic Surgery, Medical University of Vienna, Währinger Gürtel 18-20, 1090 Vienna, Austria; 7 Department of Experimental Pharmacology, National Institute of Oncology, Ráth György u. 7-9, 1122 Budapest, Hungary; tozsi@oncol.hu; 8 Department of Pathology, University Medicine Essen, Hufelandstraße 55, 45147 Essen, Germany; agnes.bankfalvi@uk-essen.de

**Keywords:** metastatic melanoma, spitzoid melanoma, targeted therapy, MEK mutation, MEK inhibitor

## Abstract

**Simple Summary:**

Spitzoid melanoma is a rare tumor type and so far preclinical models for translational research have also been also lacking. We established a cell line from a metastatic spitzoid melanoma that is, according to our knowledge, the first cell model from this tumor type. The cells carried a novel activating mutation in the region of the MEK1 protein that influences the sensitivity of the mutant protein to MEK inhibitors. We tested the cells’ sensitivity to clinically used and newly developed MEK inhibitors in both in vitro and in vivo models. The clinically approved MEK inhibitor strongly reduced both in vitro and in vivo tumor growth and might be an effective therapy for tumors with this kind of MEK mutation.

**Abstract:**

Spitzoid melanoma is a rare malignancy with histological characteristics similar to Spitz nevus. It has a diverse genetic background and in adults, a similarly grim clinical outcome as conventional malignant melanoma. We established a spitzoid melanoma cell line (PF130) from the pleural effusion sample of a 37-year-old male patient. We found that the cell line carries a rare MEK1 mutation (pGlu102_Lys104delinsGln) that belongs to the RAF- and phosphorylation-independent subgroup of MEK1 alternations supposedly insensitive to allosteric MEK inhibitors. The in vivo tumorigenicity was tested in three different models by injecting the cells subcutaneously, intravenously or into the thoracic cavity of SCID mice. In the intrapleural model, macroscopic tumors formed in the chest cavity after two months, while subcutaneously and intravenously delivered cells showed limited growth. In vitro, trametinib—but not selumentinib—and the ATP-competitive MEK inhibitor MAP855 strongly decreased the viability of the cells and induced cell death. In vivo, trametinib but not MAP855 significantly reduced tumor growth in the intrapleural model. To the best of our knowledge, this is the first patient-derived melanoma model with RAF- and phosphorylation-independent MEK mutation and we demonstrated its sensitivity to trametinib.

## 1. Introduction

Spitzoid melanocytic tumors are diagnostically challenging as a heterogeneous group ranging from the benign Spitz nevi to atypical Spitz tumor and spitzoid melanomas. Apart from histological characteristics such as large epithelioid to spindled-shaped melanocytes, recently genetic alternations were also identified that frequently occur in Spitz tumors which are distinct from those occurring commonly in cutaneous melanomas. In Spitz tumors, mutations of HRAS (15%), inactivation of BAP1 (5%) and kinase fusions of ALK, ROS1, NRTK1, RET, MET and BRAF (50%) were described [1]. Usually, one of these mutations is already present in the Spitz nevi, initiating rapid growth. As other genetic aberrations are added, the tumor-suppressor mechanisms become dysfunctional, leading to the formation of atypical Spitz tumors and a spitzoid melanoma with metastatic capacity can develop [2]. According to the 2018 WHO classification, spitzoid melanomas are classified not only by morphological but also the above described genetic characteristics [3]. In cutaneous melanoma, four subtypes were identified based on their mutational backgrounds. The BRAF subtype (~50%), which mostly carries a BRAF V600E mutation. The RAS subtype (~25%), which includes mostly NRAS point mutations or rarely KRAS and HRAS hot-spot mutations. In the NF1 subtype (~10%) the tumor-suppressor NF1 is inactivated, which is a negative regulator of RAS. The remaining tumors belong to the “triple wild type subtype” [2]. In a recent study, 25 spitzoid melanomas were analyzed and in 36% Spitz-tumor-specific mutations were found, while in 50%, mutations activating the MAP-kinase pathway were present [3]. Another study found MAPK pathway effecting mutations [4] in 66% of 27 spitzoid melanoma samples. Furthermore, RNA and DNA sequencing of 80 Spitz tumors (ST), 26 Spitz melanomas (SM) and 22 melanomas with spitzoid features (MFS) showed that in STs and SMs, kinase fusions were prevalent, while in MSFs, mostly BRAF, NRAS and NF1 mutations were present [5]. These results indicate that spitzoid melanomas are a genetically diverse group despite their histological similarity.

Aberrant signaling of the MAPK pathway is very common in cancer, leading to dysregulated cell proliferation and differentiation. The RAS and RAF oncogenes are mutated most frequently, while alterations in the MEK genes are rarely present. MEK1 and 2 are dual-specificity protein kinases that activate ERK [6]. ERK has numerous substrates and its increased activity was described in up to 80% of all cancers. The overall similarity between MEK1 and MEK2 is 80%; however, they still significantly differ in their protein partners and kinase activity [7]. MEK1 becomes activated when it is phosphorylated in its activation loop (S218 and S222), which initiates the opening of the catalytic pocket. Its inactivation is facilitated by PPA2, which dephosphorylates these two sites. Furthermore, a negative regulatory region in the N-terminus (helix A) interacts with residues in the kinase domain and stabilizes the inactive conformation of the enzyme [6,8]. This results in a low basal activity of this kinase. It was demonstrated that deletions or mutations in these regulatory regions can alter the activation state of MEK [9]. In human tumors, MEK mutations are relatively rare (< 1%) [10] but they were identified in several cancer types such as lung cancer, ovarian cancer, colorectal cancer and melanoma [11,12,13,14]. A recent study suggested that MEK1 mutations can be categorized into three groups based on their activation mechanisms [15]. RAF-dependent MEK mutations only amplify RAF signaling since they alone cannot drive ERK phosphorylation. They are almost always associated with other RAS or RAF mutations and further increase ERK activation in the tumor cells. They are also sensitive to ERK-dependent feedback. The second group consists of RAF-regulated mutants that have various levels of basal activity but through phosphorylation by RAF, their activation is further increased. These mutations are also often present together with other upstream MAPK mutations and are sensitive to ERK-related feedback. Several of these were described as acquired-resistance mutations after upstream inhibitor treatment [10]. The third group contains MEK1 mutants that are constitutively active and their activation is RAF- and phosphorylation-independent. These mutants typically carry an in-frame deletion in the kinase domain between amino acids 98 to 104. This region is necessary for the binding of BRAF and it is also a negative regulator of MEK kinase activity.

Trametinib and selumetinib are allosteric inhibitors of MEK1 and MEK2. Trametinib is an FDA-approved drug for unresectable or metastatic melanoma, while selumetinib is FDA-approved as an orphan drug for stage III and IV differentiated thyroid cancer [16] and as a treatment for neurofibromatosis type 1 and inoperable plexiform neurofibromas [17]. They bind to the allosteric pocket next to the catalytic site and they preferentially bind to the inactive form of the proteins. It was found that RAF-independent MEK mutants are less sensitive to allosteric MEK inhibitors supposedly because they are constitutively in the active form [15]. ATP-competitive MEK inhibitors bind to the catalytic region of the enzyme and were shown to be effective to overcome BRAF-inhibitor-induced resistance [18,19]. MAP855 is a recently developed ATP-competitive MEK inhibitor and Gao et al. found that RAF-independent MEK mutants are sensitive to this drug.

In the current study, we established a new cell line from a patient diagnosed with spitzoid melanoma. In the tumor cells, we identified a unique deletion and insertion alternation in MEK1 within the region that was affected in RAF-independent MEK mutants. We treated the cells with two allosteric and one ATP-competitive MEK inhibitor and compared their effects both in in vitro and in vivo experiments.

## 2. Results

### 2.1. Patient History

**Figure 1:**
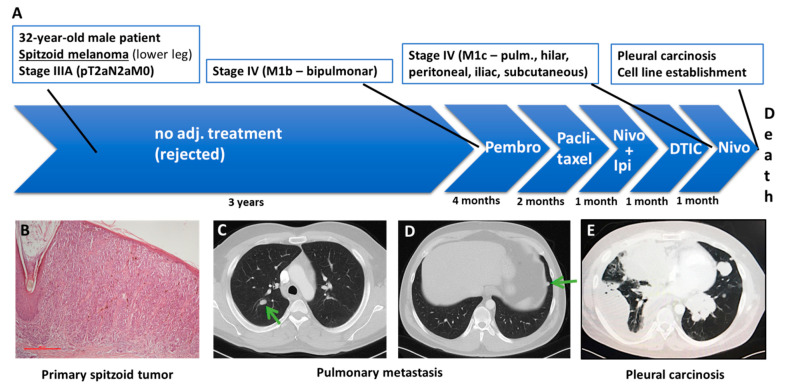
The patient’s treatment course. (**A**) The patient was diagnosed with a spitzoid melanoma stage IIIA (pT2aN2aM0). Three years later, bipulmonary metastases were identified. Subsequently, the patient received several lines of treatment including pembrolizumab, paclitaxel, a combination of nivolumab and ipilimumab and dacarbazine (DTIC). (**B**) Paraffin-embedded section stained with hematoxylin–eosin from the primary spitzoid tumor. The scalebar represents 500 µm. Computed tomography (CT) scans of the patient demonstrating pulmonary metastases on the right (**C**) and on the left (**D**) side of the lung. (**E**) CT scan of the patient showing pleural carcinosis and malignant pleural effusion on the right side. The PF130 cell line was established from the pleural effusion.

A 32-year old man was diagnosed with a spitzoid melanoma of the left lower leg with a tumor thickness of 1.7 mm. Two sentinel lymph nodes were positive, resulting in an initial disease stage IIIA pT2aN2aM0 (Figure 1). The examination of the BRAF and NRAS genes did not reveal any mutation. The patient did not receive adjuvant treatment. Two years after the initial diagnosis, metastases were detected in the left iliac lymph nodes and the disease progressed to stage IV. Tissue from one of the metastatic lymph nodes was analyzed with a 30-gene NGS panel used for melanomas; however, no mutation was identified. The PD-L1 status of the tumor was negative. The patient refused adjuvant low-dose interferon therapy. One year later, bipulmonary metastases developed and the patient underwent several lines of systemic therapies including treatment with anti-PD-1 antibody pembrolizumab for four months, paclitaxel for two months, combination checkpoint inhibition with nivolumab and ipilimumab for one month and dacarbazine (DTIC) also for one month and nivolumab monotherapy for 1 month. Every line of therapy was stopped due to tumor progression. As the malignancy advanced, the patient developed pleural carcinosis on the right side, leading to pleural effusion. A video-assisted thoracoscopy (VATS) was performed and an indwelling pleural catheter (PleurX) was inserted in order to treat the effusion. Shortly after this procedure, the patient died. The PF130 cell line was established from the pleural effusion sample of the patient.

#### 2.1.1. Identification of a Novel Rare MEK1 Mutation

To investigate if a driver mutation in the PF130 cells was present, we performed NGS analysis. Importantly, we found a mutation in the *MAP2K1* gene (303_310del8insGln) resulting in a deletion of the amino acids Glu102_Lys104 (ΔE102-K104) and insertion of a Gln. This region of MEK1 was described as a mutational hot spot in various cancer types, including cutaneous melanoma [20]. In a study which compared the mechanism of action of different MEK1 mutants, it was demonstrated that MEK1 mutants in the ΔL98-I103, ΔE102-I103, ΔI99-K104, and ΔI103-K104 regions are constitutively active and independent from the phosphorylation by RAF [15]. The mutation was already present in the primary spitzoid tumor and in the iliac lymph node metastasis. We found no TERT promoter, ALK kinase fusion (exons 20–25) or BAP1 mutation in the cell line. The results of the mutational analysis performed on the PF130 cell line and on the tumor tissue are summarized in Table S1.

#### 2.1.2. In Vitro Sensitivity to Allosteric and ATP-Competitive MEK Inhibitors

**Figure 2:**
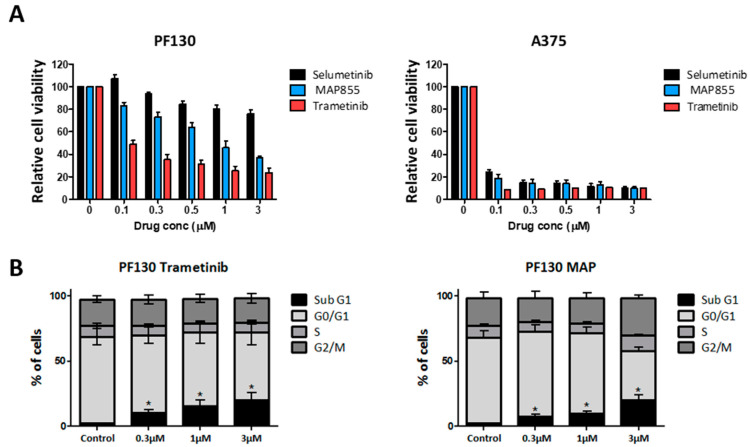
Sensitivity of PF130 cells to different MEK inhibitors. (**A**) Cell viability was measured after treatment with the MEK inhibitors selumetinib, trametinib or MAP855 for 72 hours. (**B**) Cell cycle analysis was performed after treatment with trametinib or MAP855 for 72 hours. Statistical comparison was calculated by one-way ANOVA with Dunn´s multiple comparison test (*p* < 0.05). Bars represent means ± SE from two to three independent experiments.

It was described earlier that deletions within the ^98^LIHLEI^104^K region of MEK1 can lead to constitutive and RAF-independent activation of MEK1 and they strongly influence the sensitivity to different MEK inhibitors. We treated the cells with two types of allosteric MEK inhibitors (selumetinib and trametinib) and one newly developed ATP competitive MEK inhibitor (MAP855). We performed a viability assay with all three drugs and found that selumetinib had little effect, while both MAP855 and trametinib strongly decreased the viability of PF130 cells in a concentration-dependent manner (Figure 2A). In addition, trametinib treatment was significantly more effective (IC50: 59 nM) than MAP 855 treatment (IC50: 1.13 µM). As a reference, we used the A375 melanoma cell line that carries a BRAF V600E mutation. In this cell line, all three treatments strongly decreased the ratio of viable cells already in the lowest concentration.

We also investigated how MEK inhibition influences the cell cycle of PF130 cells (Figure 2B). We found that both trametinib and MAP 855 treatment substantially increased the ratio of the cells in the Sub G1 phase but did not significantly alter the ratio of the cells in the other cell cycle phases, suggesting that both treatments were able to induce cell death in a portion of the cells.

**Figure 3:**
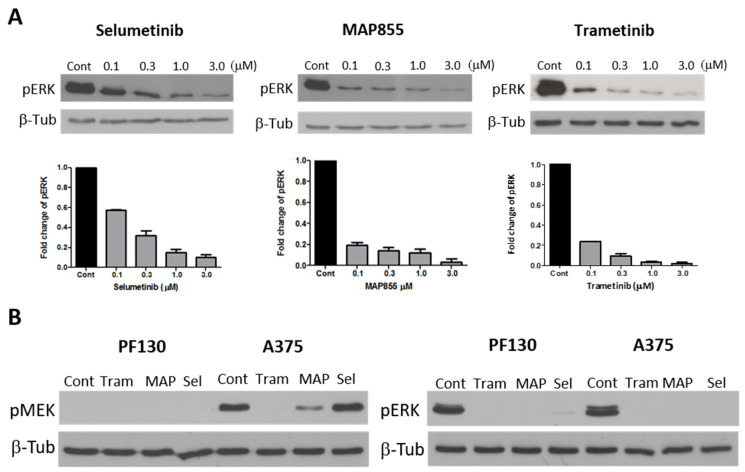
ERK activation and MEK phosphorylation after MEK-inhibitor treatments. (**A**) ERK activation was analyzed by Western blot in PF130 cells after treatment with the MEK inhibitors selumetinib, trametinib or MAP855 for 48 hours. After densitometric analysis, changes in pERK level were normalized to the expression levels of β-tubulin and expressed as fold change compared to untreated controls. Bars represent means ± SE from three independent experiments. (**B**) MEK phosphorylation (SER217/221) was measured after treatment with trametinib (0.3 µM), MAP855 (0.5 µM) or selumetinib (0.5 µM) for 2 hours. Whole blot can be found at Figures S2 and S3.

We also analyzed the effect of all three MEK inhibitors on ERK activation as it is a direct target of MEK1 (Figure 3A). Interestingly, ERK phosphorylation was strongly decreased by both MAP855 and trametinib at all concentrations and by selumetinib in a concentration-dependent manner. We also analyzed the phosphorylation of MEK at the phosphorylation sites by RAF kinases and found that these sites were not phosphorylated in the PF130 cells (Figure 3B), even without treatment. This is in line with an earlier study finding that MEK1 mutants with a deletion in this region are constitutively active independently from their phosphorylation by RAF kinases on these sites. In A375 cells, MEK phosphorylation was decreased by both trametinib and MAP 855 treatments but not by selumetinib (Figure 3B, Figure S1).

#### 2.1.3. In Vivo Tumorigenicity of PF130 Cells

**Figure 4:**
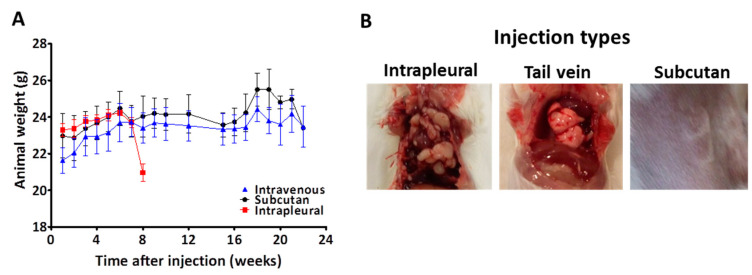
In vivo tumorigenicity of PF130 cells. (**A**) Cells were injected into female SCID mice subcutaneously (*n* = 3), into the tail vein (*n* = 4) or intrapleural (*n* = 3). The weight of the animals was measured once a week. (**B**) The injection into the thoracic cavity led to robust tumor growth within two months. In contrast, tail vein and subcutaneously injected cells survived but the tumor load was minimal even after six months.

First, we analyzed the tumor-forming capacity of PF130 cells in three different models. The same amount of cells was injected into female SCID mice subcutaneously (*n* = 3), into the tail vein (*n* = 4) or into the thoracic cavity (*n* = 3). The weight of the animals was measured once a week. After 8 weeks, the weight of the mice with intrathoracic injection rapidly declined (Figure 4A), the animals were sacrificed, and we found that in the chest cavity, macroscopic tumors were present (Figure 4B). Animals belonging to the other two groups presented a steady weight and survived for six months (Figure 4A), than they were sacrificed. In these animals, we could also identify smaller tumors on the surface of the lung in both the tail vain and subcutaneously injected model (Figure 3B). 

#### 2.1.4. Trametinib Is Effective Against the Novel MEK1 Mutant Cells In Vivo

**Figure 5:**
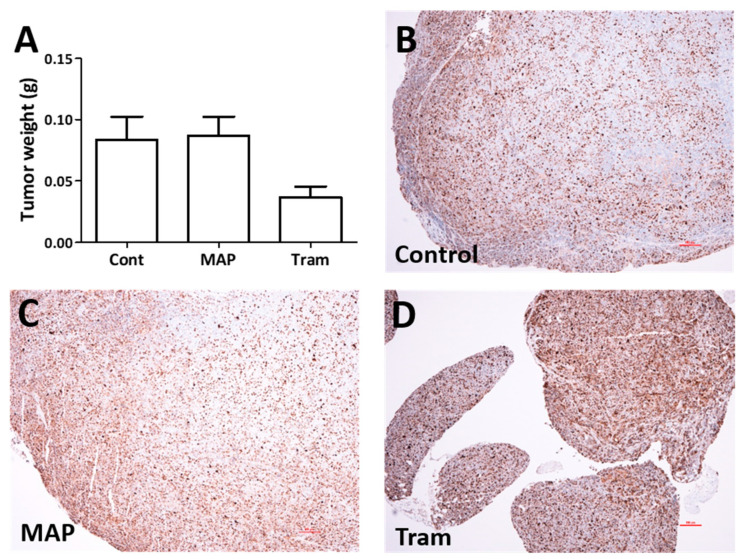
Effect of MEK inhibition on in vivo tumor growth. Cells were injected into the thoracic cavity of SCID mice and 6 weeks later, the animals were treated with trametinib (2 mg/kg) or MAP 855 (15 mg/kg) on three weekdays (two days apart) for three weeks. (**A**) The weight of the dissected tumor nodules at the end of the experiment. The one-way ANOVA test indicated a significant difference (*p* = 0.0327). (**B**–**D**) Ki67-stained tumor nodules show that in the small nodules and in the periphery of the large tumor nodules, up to 90% of the cells are labelled. While in control or MAP855-treated animals, the nodules are often growing far beyond a diameter of 1 mm—we rarely observed such large nodules in the trametinib-treated animals. The scalebars represent 100 microns.

Since both trametinib and MAP855 decreased cell viability effectively and could induce cell death in PF130 cells, we tested the efficacy of these compounds in vivo. We injected the cells into the thoracic cavity of SCID female mice and waited 6 weeks for tumor formation. Then, the animals were divided into three groups (*n* = 9), control, trametinib-treated (2 mg/kg) and MAP855-treated (15 mg/kg) and the drugs or the vehicle were administered every two days for three weeks. We found that trametinib treatment significantly decreased tumor growth (Figure 5), while MAP855 treatment had no effect. These results show that trametinib can inhibit the growth of PF130 cells effectively both in vitro and in vivo.

## 3. Discussion

In our work, we established a novel cell line, PF130, which to the best of our knowledge, is the first spitzoid-melanoma-derived cell line. Spitzoid melanoma is a type of melanoma that has similar histological characteristics to the Spitz nevus. While in children it has a more favorable prognosis, in adults, no difference was found in the mortality rate of patients with spitzoid or conventional malignant melanoma [21]. It is a rare tumor type, thus it is particularly important to generate cell models to test both in vitro and in vivo novel therapeutic modalities. In this patient with a spitzoid malignant melanoma of the left lower leg, a unique MEK1 mutation in the PF130 tumor cells was found. Interestingly, this mutation was already present in the primary tumor tissue. This MEK1 mutation caused a deletion of three amino acids (glutamic acid (102)-isoleucin (103) –lysine (104)) and an insertion of a glutamine. The ΔE102 - K104 deletion mutation in MEK1 was described previously in a spitzoid melanoma patient together with a HRAS hotspot mutation [3]. We found no HRAS mutation in the PF130 cells.

The deleted residues in MEK1 are located in the kinase domain and play an important role in the autoinhibition of the enzyme. Deletions within the ΔL98-K104 area of MEK1 were found in cutaneous melanoma, lung adenocarcinoma and colon carcinoma tumor tissues [22] and the ΔE102-I103 alteration was present most frequently. Gao et al. [15] characterized seventeen MEK1 mutants, including four that effect the above-mentioned region (ΔL98-I103, ΔE102-I103, ΔI99-K104, and ΔI103-K104) after cloning and expression in 293H cells. They found that these four strongly induced both ERK and MEK phosphorylation in the cells and mutation of the RAF phosphorylation sites S218 and S222 to alanine in these mutants did not decrease their ERK activation capacity. Furthermore, when these mutations were introduced into RAF-less cells, they were able to form tumors in nude mice. This is in accordance with our findings in PF130 cells, where we also found a very high level of pERK activation but without MEK phosphorylation on the S218 and S222 sites. Furthermore, PF130 was able to form tumors in SCID mice in three different tumorigenicity models. Gao et al. treated three out of the four mutants with two allosteric MEK inhibitors PD0325901 and trametinib and found that the two mutants where six amino acids were deleted (ΔL98-I103, ΔI99-K104) were resistant to the treatments and pERK activation did not decrease. However, treatment with the novel ATP-competitive MEK inhibitor MAP855 decreased ERK phosphorylation even in these mutants and all others in the 300–3000 nM range. In case of the third type of mutant where only two amino acids were deleted (ΔE102-I103), both trametinib and MAP855 reduced ERK activation in the 100–1000 nM range, while PD0325901 had a weaker effect. We found that PF130 cells reacted to the MEK-inhibitor treatment similarly to this third mutant because both trametinib and MAP855 were able to decrease ERK activation of the cells in the 100–1000 nM range. Since allosteric MEK inhibitors bind preferentially to the inactive form of the enzyme, they supposedly cannot bind to the mutants lacking the whole 98–104 region, while they are constitutively in the active form. The deleted region is shorter in the aforementioned third mutant and in the PF130 cells and that might cause only the partial activation of these mutants, making it possible for allosteric inhibitors to bind to them. This is in line with previous findings in other kinases with similar structure, such as EGFR, HER2 or BRAF [23]. Furthermore, in PF130 cells, the deletion of the three amino acids is accompanied by an insertion of glutamine and this might further influence the activation state of the enzyme. Of note, in the RAF-dependent and RAF-regulated mutants, trametinib had a strong inhibitory effect at a concentration as low as 10 nM.

We also demonstrated that both trametinib and MAP855 treatment strongly decreased the viability of the cells and induced cell death. Trametinib was described to decrease the number of cells in the synthesis phase in BRAF-mutant lung cancer cells [24], and to induce G1 cell cycle arrest and apoptosis in RAS- and RAF-mutated melanoma cells [25,26]. In PF130 cells, trametinib strongly increased the ratio of the cells in the subG1 phase but it had only a subtle effect on the portion of proliferating cells. MAP855 had a similar effect but a higher concentration of the drug was required both to decrease the viability of the cells and to induce apoptosis. Interestingly, when we analyzed the effect of both drugs in an in vivo experiment, only trametinib was effective. We inoculated PF130 cells into the thoracic cavity of female SCID mice and waited 6 weeks for tumor formation. We chose to use this model system instead of subcutaneous or tail vein injection as the tumorigenicity of the cells was much stronger intrapleurally. This might be due to fact that the cell line was established from the pleural effusion of the patient and already adapted to this environment. The animals were treated with trametinib (2 mg/kg) and MAP855 (15 mg/kg) or the vehicle every two days for three weeks. Trametinib was able to significantly reduce the size of the tumors in the mice in good accordance with previous findings in melanoma [27] and colorectal cancer cell lines [28]. However, MAP855 treatment did not have a significant effect at this concentration. In a recent study, MAP855 was successfully used to inhibit ERK activation in MEK1 V211D-mutant colon cancer cells that were already resistant to binimetinib and MAP855 treatment decreased tumor growth by 30% through reduction of proliferation and induction of apoptosis in the in vivo PDX model of the original patient tumor [29]. In the colon cancer study, a higher (30 mg/kg) concentration was used, indicating that a higher treatment concentration could have increased the efficacy in our experiment as well.

## 4. Materials and Methods

### 4.1. Cell Lines and Compounds

The PF130 cell line was established from a malignant pleural effusion. Briefly, the sample was centrifuged at 1200× *g* for 10 min, then the supernatant was removed. The pellet was resuspended in RPMI1640 containing 10% FBS, and 100 U/mL penicillin–streptomycin and the suspension was seeded on a 25 cm^2^ tissue culture flask. The adherent cells were cultured for 10 passages to obtain a tumor cell culture free of non-malignant cells before experiments were initiated. The study was approved by the Ethics Committee at the University Hospital Essen (#18-8208-BO) and written consent from the patient was obtained. The A375 cell line was purchased from ATCC. Both cell lines were subjected to Single Nucleotide Analysis by Multiplex Cell Line Authentication (Multiplexion, Heidelberg, Germany). Both cell lines were cultured in DMEM containing 10% FBS and 1% penicillin–streptomycin at 37 °C and 5% CO_2_ in a humidified atmosphere. Selumetinib and trametinib were purchased from Selleck Chemicals (Houston, TX, USA), and were dissolved in DMSO at 10 mM concentration and stored at −80 °C in aliquots. MAP855 was obtained from Novartis and dissolved in DMSO.

### 4.2. Viability Assay

Cell viability was analyzed with Sulforodamine B (SRB) assay based on previously described protocols [30]. Briefly, 5000 cells were seeded on the inner 60 wells of a 96 well-plate. Twenty-four hours later, medium was applied with various drug concentrations. Seventy-two hours later, the treatment was stopped by fixation with 10% TCA. Then, SRB dye was applied for 15 minutes, Afterwards, excess dye washed out with 1% acetic acid the protein-bound dye was dissolved with 10 mM Tris buffer. Optical density was measured with a microplate reader (EL800, BioTec Instruments, Winooski, VT, USA) at 570 nm. Measurements were carried out in triplicate and in three biological replicates. IC50 values were calculated with CompuSyn software (ComboSyn Inc, Paramus, NJ, USA).

### 4.3. Cell Cycle Assay

The percentage of cells in the different cell cycle phases was defined based on their DNA content. After the mixing of the lysis buffer (Solution 10, 910-3010, Chemometec, Lillerød, Denmark) with the DAPI stain solution (Solution 12, 910-3012, Chemometec), cells were trypsinized and incubated with the lysis buffer for 5 minutes at 37 °C. Then, the reaction was stopped by adding the stabilization buffer to the solution (Solution 11, 910-3011, Chemometec). Cellular fluorescent was analyzed with NucleoCounter NC-3000system (Chemometec).

### 4.4. Western Blot Analysis

Cells were seeded on 6-well plates (1–2 × 10^5^ cells/well) and 24 h later, the medium was changed and the treatment was applied for 48 hours. Afterwards, cells were washed twice with PBS and 6% TCA was added to precipitate all protein. After centrifugation (8000 rpm, 10 min, 4 °C), the pellet was dissolved in electrophoresis sample buffer (62.5 mM Tris–HCl, pH 6.8, 2% SDS, 10% glycerol, 5mM EDTA, 125 mg/mL urea, 100mM dithiothreitol) and samples were loaded in equal amounts (30 μg) on 10% acrylamide gels. Rabbit monoclonal anti-phospho-p44/42MAPK (ERK1/2) (Cell Signaling, 4370S, dilution 1:2000), rabbit monoclonal anti-phospho-MEK1/2 (Ser217/221) (41G9) (Cell Signaling, 9154, 1:1000) and rabbit polyclonal anti-beta-tubulin (Abcam, ab6046, 1:2000) primary antibodies were used for immunostaining. As secondary antibody, HRP-conjugated anti-rabbit secondary antibodies (Jackson ImmunoResearch, dilution 1:10,000) were used and for detection, Pierce ECL Western Blotting Substrate (Thermo Scientific, Waltham, MA, USA) was applied followed by luminography.

### 4.5. In Vivo Tumorigenicity Assay

The tumor-forming capacity of PF130 cells was analyzed in three different models. We injected one million cells in 200 microliter serum free DMEM into female SCID mice subcutaneously (*n* = 3), into the tail vein (*n* = 4) or intrapleural (*n* = 3). The weight of the animals was measured once a week. When animals lost more than 15% of their weight or after six months, mice were sacrificed by cervical dislocation and macroscopic tumor nodules were collected, fixed in 4% paraformaldehyde and paraffin-embedded (FFPE) tissue blocks were prepared. The animal model protocol was performed following the Guidelines for Animal Experiments and was approved for the Department of Experimental Pharmacology in the National Institute of Oncology, Budapest, Hungary (PEI/001/2574-6/2015).

### 4.6. MEK Inhibitor Treatment in the Pleural Carcinosis Model

One million PF130 cells were inoculated in 200 μL serum free DMEM into the thoracic cavity of SCID female mice and waited 6 weeks for tumor formation. The weight of the animals was measured once a week. Then, the animals were divided into three groups (*n* = 9), control, trametinib (2 mg/kg) treated and MAP855 treated (15 mg/kg) and the drugs or the vehicle were administered and the animals weighed every Monday, Wednesday and Friday for three weeks. MAP855 and trametinib were dissolved in solvent containing 5% 1.0N HCl, 42% PEG300 and 20% solutol. At the end of the experiment, after opening up the chest, we thoroughly searched the whole thoracic cavity (including the surface of the pericardium and diaphragm) for tumor nodules and dissected them out. We pooled and weighed the nodules for each animal separately and fixed them in formalin. After embedding in paraffin, sections were stained with H&E as well as immunohistochemically labeled with ki67 (clon 30-9; monoclonal mouse IgG, Roche-Ventana) antibody following standard protocols on the Ventana BenchMark Ultra system (Roche Tissue Diagnostics, Grenzach-Vyhlen, Germany). The animal model protocol was performed following the Guidelines for Animal Experiments and was approved for the Department of Experimental Pharmacology in the National Institute of Oncology, Budapest, Hungary (PEI/001/2574-6/2015).

## 5. Conclusions

We established the—to the best of our knowledge—first patient-derived spitzoid melanoma cell model that carries a novel RAF- and phosphorylation-independent MEK mutation. Our study suggests that certain allosteric or ATP-competitive MEK inhibitors might be effective in tumors driven by this unique class of MEK mutations.

## Data Availability

Upon reasonable request all data and commercially not available materials can be obtained from the corresponding author.

## Supplementary Materials

The following are available online at https://www.mdpi.com/2072-6694/13/4/829/s1, Table S1: Mutational analysis performed on PF130 cell line, Figure S1: ERK activation and MEK phosphorylation after MEK inhibitor treatments, Blots showing all the bands with molecular weight markers for Figure 3A (Figure S2) and 3B (Figure S3).
