# Supplementary material for: Allosteric and ATP-Competitive MEK-Inhibition in a Novel Spitzoid Melanoma Model with a RAF- and Phosphorylation-Independent Mutation
Source: Cancers (Basel). 2021 Feb 16;13(4):829. doi: 10.3390/cancers13040829 (PMC7920251; doi:10.3390/cancers13040829)
Supplement: Supplementary file 1 [file cancers-13-00829-s001.pdf]

Supplementary Material

Allosteric and ATP-Competitive MEK-Inhibition in a Novel Spitzoid Melanoma Model with a RAF- and Phosphorylation-Independent Mutation

Luca Hegedüs, Özlem Okumus, Elisabeth Livingstone, Marcell Baranyi [3], Ildikó Kovács, Balázs Döme, József Tóvári, Ágnes Bánkfalvi, Dirk Schadendorf, Clemens Aigner and Balázs Hegedüs

Table S1. Mutational analysis performed on PF130 cell line. Gene mutational status was analyzed by NGS. TERT promoter and ALK mutation were analyzed with Sanger sequencing.

Gene	PF130 cell line
**MAP2K1**	**c.303_310del8insGln**
BRAF	WT
GNAQ	WT
HRAS	WT
TERT	WT
PTEN	WT
MAP2K2	WT
MITF	WT
SMARCA4	WT
CTNNB1	WT
BAP1	WT
NRAS	WT
GNA11	WT
RAC1	WT
CDKN2A	WT
TP53	WT
PIK3R1	WT
ARID2	WT
EZH2	WT
FBXW7	WT
SF3B1	WT
KIT	WT
NF1	WT
CDK4	WT
PIK3CA	WT
ARID1A	WT
IDH1	WT
KRAS	WT
TERT promoter	WT
ALK (Exone 20-25)	WT

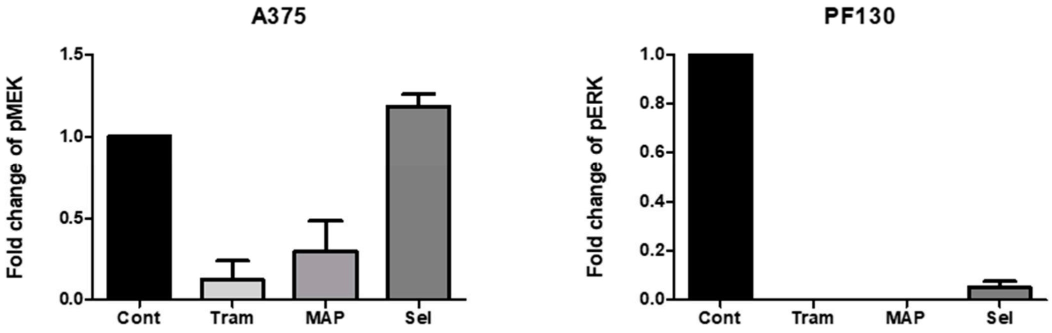

Figure S1. ERK activation and MEK phosphorylation after MEK inhibitor treatments**.** MEK phosphorylation (SER217/221) and ERK activation were measured after treatment with trametinib (0.3 μM), MAP855 (0.5 μM) or selumetinib (0.5 μM) for 2 hours. Densitometric analysis of pMEK and pERK levels were normalized to the expression levels of β-tubulin and expressed as fold change compared to untreated controls. Bars represent means ±SE from three independent experiments.

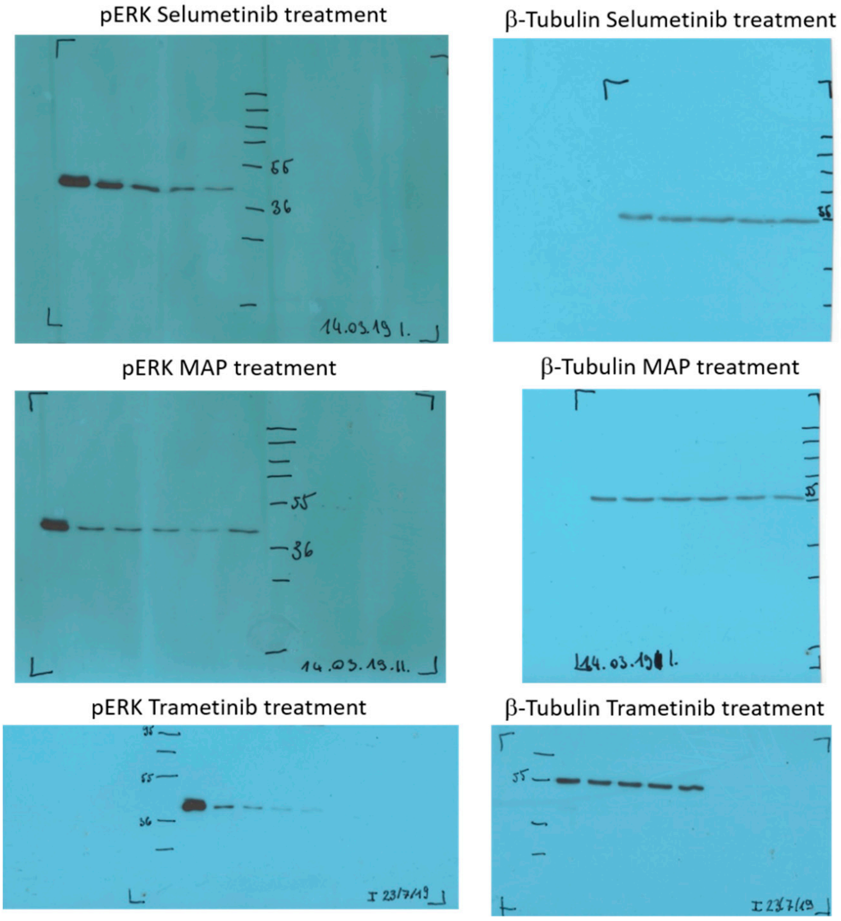

Figure S2. Blots showing all the bands with molecular weight markers for figure 3A. PVDF membranes were cut in the line of the 70kDa marker before staining with β-Tubulin antibody.

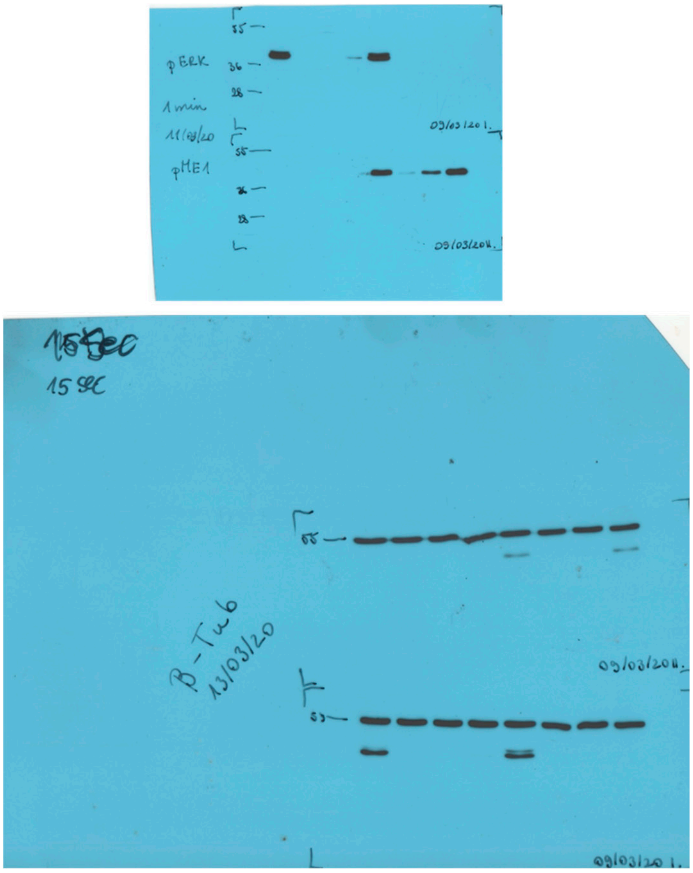

Figure S3. Blots showing all the bands with molecular weight markers for figure 3B. PVDF membranes were cut in the line of the 70kDa marker before staining with pERK, pMEK or β-Tubulin antibodies.
